# Worldwide Prevalence and Risk Factors of *Helicobacter pylori* Infection in Children

**DOI:** 10.3390/children9091359

**Published:** 2022-09-06

**Authors:** Reka Borka Balas, Lorena Elena Meliț, Cristina Oana Mărginean

**Affiliations:** Department of Pediatrics I, “George Emil Palade” University of Medicine, Pharmacy, Science and Technology of Târgu Mureș, Gheorghe Marinescu Street No 38, 540136 Târgu Mureș, Romania

**Keywords:** *Helicobacter pylori* infection, worldwide, children

## Abstract

*Helicobacter pylori* is usually acquired during childhood. The reports from the last two decades pointed out a decrease in *H. pylori* prevalence across geographical areas worldwide compared to previously reported data. Most of the studies performed in America found an overall *H. pylori* infection prevalence of approximately 50%. The most important risk factors in America include being male, poor adherence or difficult access to treatment, and the lack of in-home water service. Despite the descending trend in prevalence worldwide, the overall prevalence in Africa remains very high (70%). Nevertheless, the prevalence of *H. pylori* in children without gastrointestinal who underwent screening was reported to be only 14.2%. The main risk factors in Africa are having a traditional pit or no toilet, poverty, birth order, source of drinking water, or being a farmer. Asia seems to have the widest variations in terms of *H. pylori* prevalence. Several risk factors were reported in Asia to be associated with this infection, such as lower income and educational level, house crowding, rural residence, ethnicity, the use of tanks as water supplies, alcohol drinking, active smoking, eating spicy food or raw uncooked vegetables, poor living conditions and sanitation. The overall prevalence of *H. pylori* infection in European children is almost 25%. Portugal has the highest prevalence of all European countries at 66.2% in children 13 years of age. The risk factors in European individuals consist of living in rural areas, eating unwashed fruits and vegetables, not washing hands after school, low parental education and unemployment, and short education duration. Further studies are required to identify the precise mechanisms involved in the discrepancies of *H. pylori* prevalence worldwide.

## 1. Introduction

*Helicobacter pylori* (*H. pylori*) is commonly acquired during childhood. Its long-term persistence might lead to gastric adenocarcinoma and mucosa-associated lymphoid tissue lymphoma in adulthood [1]. Thus, *H. pylori* is one of the most important leading infectious causes of cancer worldwide, as 8 in 10 gastric cancers in adults are attributable to this infection [2]. Several factors which contribute to the discrepancies in terms of prevalence were reported to increase the risk of *H. pylori* infection, such as older age, hygiene conditions, a large number of family members, having a mother, a sibling or siblings infected with *H. pylori*, room or bed-sharing, drinking unboiled or non-treated water, and low socioeconomic status [3,4,5,6,7]. It was also hypothesized that prenatal transmission from infected mothers or the transmission during delivery might also contribute to the increased *H. pylori* prevalence in pediatric patients [8]. Nevertheless, most of the studies proved that this route of transmission is unlikely since no traces of *H. pylori* DNA were encountered in the placenta of pregnant women who tested positive for this infection [9]. A more recent study that assessed *H. pylori* status in mother-newborn couples proved that none of the children born to infected mothers tested positive for this infection despite a follow-up until the age of three months [10]. The prevalence of this infection is difficult to accurately assess based on the common lack of symptoms in small age groups. Thus, only 5% of the infected children develop gastritis, ulcer disease, or other *H. pylori*-related gastrointestinal such as extraintestinal disorders, including growth retardation, iron refractory iron deficiency anemia, or thrombocytopenia [6,11]. Among the few studies performed with this aim, a study from 2017 reported a global childhood prevalence of 33%, but without assessing the differences between geographic areas, countries, or sub-regions [12]. Contrariwise, in adults, the prevalence was noticed to vary widely, from 24% to 73% across different populations, with a pooled global prevalence of approximately 50% [13]. Taking into account that the infection is usually acquired during childhood and also that the prevalence presents wide variations, we state the following hypotheses: (1) in the areas with low prevalence, we might be talking about a low infection rate during childhood; (2) despite the increased infection rate in childhood, the eradication regimens prove to be extremely efficient and can completely eradicate the infection until adulthood. 

Most of the studies during the last decades underlined a descending trend of *H. pylori* prevalence across geographical areas worldwide, estimating that the prevalence of this infection in pediatric subjects decreased by one-third, from 39% before 2000 to 26% in 2010 [14]. A possible explanation for this decrease might be related to the worldwide improvements in socioeconomic status, but also to environmental and living conditions resulting in better sanitation and decreased transmission over time [14]. Nevertheless, we should be aware that most cases of *H. pylori* infection during childhood are asymptomatic and therefore are not tested and/or diagnosed. One of the main challenges related to *H. pylori* eradication is to diagnose the infection as soon as possible with increased accuracy. Therefore, choosing the proper diagnostic method for this infection might seem simple, but the choice should be based on several criteria related to the patient’s age and geographical peculiarities [15]. Taking into account that all children should be treated regardless if they present symptoms or not [16,17], choosing the most effective and accurate diagnostic method might severely influence the success of eradication and might increase the frequency of *H. pylori*-related complications during adulthood. According to the Maastricht V/Florence Consensus Report, the following adult groups should benefit from *H. pylori* testing: subjects with peptic ulcers, especially those receiving aspirin or non-steroidal anti-inflammatory drugs, but also those with a history of peptic ulcers; individuals with dyspepsia originating from high prevalent areas; subjects with gastritis focusing in particular on those who were administered long-term proton pump inhibitors; patients with localized early stage MALToma, gastric cancer, or increased risk of gastric cancer; but also those with thrombocytopenic purpura, vitamin B_12_, and iron deficiency without an identifiable cause [18]. Regarding pediatric patients, the recent guidelines of the European and North American Societies of Pediatric Gastroenterology, Hepatology, and Nutrition recommended against a ‘test and treat’ strategy for *H. pylori* infection in this age group. They stated that diagnostic testing should be performed in children with gastric or duodenal ulcers but should not be a part of the initial work-up in those with functional abdominal pain, short stature, and iron deficiency, being required only if other causes have been ruled out [19]. Nevertheless, depending on the variable prevalence of *H. pylori* infection worldwide, these recommendations might be debatable in countries where this infection is highly prevalent [20]. Extraintestinal manifestations were acknowledged as a criterion for *H. pylori* testing. They should not be overrated since they are related to the systemic subclinical inflammation associated with *H. pylori* infection with severe systemic implications regardless of the patient’s age [6,21]. Thus, aside from the extraintestinal pathologies mentioned by the Maastricht Consensus, previous studies suggested that *H. pylori* infection might be related in addition to a wide spectrum of extraintestinal disorders, such as cardiovascular disorders, stroke, anemia, glaucoma, eczema, rosacea, chronic hives, diabetes, or Alzheimer’s disease [22]. Moreover, studies that assessed the level of *H. pylori* antibodies and different cardiovascular disorders noticed that they were significantly associated with coronary artery conditions [23], but also high systolic pressure and arterial stiffness in the setting of diabetes mellitus [24]. All these findings add even more value to the successful eradication during childhood for preventing a wide range of multisystemic complications. Although one of the most common infections in pediatrics, *H. pylori* still carries a lot of mystery related to its disparities in prevalence among geographic areas, host and environmental risk factors, diagnosis, mechanisms of resistance, and persistence into adulthood. 

This review aims to assess the differences between continents in terms of *H. pylori* infection epidemiology, risk factors, symptoms, or other diagnostic-related characteristics to provide a solid basis for the development of future preventive strategies adjusted to the peculiarities of each area. 

## 2. *H. pylori* Peculiarities in the United States of America

The studies performed in the United States of America (USA) reported increased rates of morbidity and mortality related to gastric cancer [25,26]. Thus, a study performed in the Caribbean, Central, and South America stated that the age-standardized incidence rate of gastric cancer differs among these regions between males and females, with an incidence of 8.7 per 100,000 men in Caribbean America, 8.1 per 100,000 men in Central America, and 12.7 per 100,000 men in South America. For the same regions, the incidence rates were 5.1, 6.3, and 6.9 per 100,000 women, respectively [25]. In terms of *H. pylori* infection, a study performed in Latin America and the Caribbean revealed a prevalence of 62.8% before the year 2000, presenting a mild decrease to 60.2% after this period [13]. A more recent study indicated a further decrease in the prevalence of *H. pylori* in these areas, estimating an overall prevalence of 57.57% [27]. Contrariwise, other studies performed in America revealed a prevalence of this infection above the numbers reported in the study of Curado et al. [27]. Thus, Hooi et al. [13] reported a prevalence of 63.4%, while the prevalence found by Zamani et al. [28] was 59.3%. Several studies performed on Alaska Native people highlighted that gastric cancer remains the third most frequent cause of cancer-related death in this population [29,30,31], three times higher than among US whites [32]. Moreover, in this area, 75% of the people were found to have positive *H. pylori* IgG antibodies, considerably increasing their risk of developing gastric cancer [33,34] based on the close relationship between this infection and gastric carcinogenesis. Thus, the prevalence in Alaska Native people resembles that reported in developing countries rather than in the population living in other areas of the US [35,36,37,38], without a decreasing pattern, especially in rural Alaska [39]. Contrarily, the prevalence of *H. pylori* infection in Mexican children presented a major decrease in recent years [40], suggesting a possible decrease in gastric cancer incidence in the future. Still, the prevalence depends on the geographic area of Mexico since studies revealed that the prevalence of this infection in Mexican children from the state of Guerrero is higher than in those from Mexico City and Monterrey (59.6% versus 35–38%) [40,41,42,43,44] (Table 1). 

It is a well-documented fact that the risk of infection increases with age, and the prevalence increases in older individuals. This fact was also sustained in America, where the available data indicated a higher prevalence in adults than children and teenagers [27,45]. Nevertheless, the prevalence of *H. pylori* in the age group up to 20 years is higher in Latin America as compared to other worldwide countries [37]. In terms of gender-related differences, the reported data in America remain controversial. Thus, while Curado et al. found no differences in *H. pylori* prevalence across genders in Latin America and the Caribbean [27], Ibrahim et al. identified a higher prevalence in males when assessing America as a whole [46]. Similarly, a study performed on Brazilian children also revealed a 30% increased risk for boys to become infected [47]. Contrariwise, a recent study that assessed Mexican children identified a significantly higher prevalence in girls [40]. Another study on Alaska Native people revealed a strong association between *H. pylori* infection and living in a more crowded home [39]. Moreover, in the same population, the status of household family members was associated with the risk of reinfection even after the infection was successfully eradicated [48]. The same study indicated that lacking in-home water service might represent a major risk for *H. pylori* reinfection [48]. Certain factors were also proven to be associated with the persistence of infection, such as having more than three children in a household or using well instead of municipal water [47] (Table 1).

Despite the current trends that show a decrease in *H. pylori* infection prevalence worldwide [35], in Latin America and the Caribbean, a recent meta-analysis failed to prove a decreasing pattern [27]. Several factors were incriminated in this area for maintaining an increased prevalence rate, such as frequent recurrences up to 7.9 per 100 people-year, poor adherence, or difficult access to treatment [49,50,51]. Moreover, certain differences might be explained, as we mentioned in the introduction, by the diagnostic test used in different areas. Thus, a recent study underlined that practitioners in the United States do not properly follow the recommendations regarding testing before and after the therapy [52]. In addition, those who perform testing do not use the most accurate test for diagnosing *H. pylori* infection. A study involving 1.9 million health plan members revealed that serology was the most commonly used test for detecting *H. pylori* infection [53]. More recent studies also showed that out of more than 100 million individuals, 70% were tested using serology, and 4.2% turned out to be positive [54]. Although the current recommendations state that serology should be confirmed by a second test [15,55], in the previous study, only 16% were confirmed by a urea breath test and 11% by stool antigen immunoassay. Contrarywise, other studies reported gastric biopsy as the most commonly used diagnostic method in more than 50% of the individuals, followed by stool antigen in 20% [48]. Therefore, these differences in terms of diagnostic tools might be responsible, at least partly, for the differences in *H. pylori* prevalence among different areas of the United States. The Houston Consensus Conference on Testing for *H. pylori* stated recommendations to help US practitioners in their clinical practice [52]. The most important topics of this Conference include the identification of appropriate patients for testing, appropriate methods for confirming the infection and for eradicating the bacterium, the effects of antibiotic susceptibility on both testing and treatment, and relevant health system particularities [52].

Nevertheless, these recommendations may vary depending on the infection prevalence in different populations. Thus, a study that included Alaska Native people proved that serology and urea breath tests were comparable in terms of accuracy when the individual had not been previously treated for *H. pylori* infection [39]. In terms of pediatrics, a study performed on Brazilian children using a urea breath test revealed an increased incidence in those living in a low-income urban community in northeast Brazil with a homogeneous socioeconomic level [56]. Moreover, another study on the same population concluded that the specificity of the urea breath test reaches up to 100% for diagnosing *H. pylori* infection in Brazilian children [57]. Another cohort of children from Mexico was mainly detected with *H. pylori* based on stool antigen, which was the most feasible method to detect this infection, even in asymptomatic children [40]. Still, detecting *H. pylori* infection in children remains challenging since most are either asymptomatic or present unspecific symptoms such as abdominal pain, vomiting, nausea, or diarrhea [58]. 

## 3. *H. pylori* Peculiarities in Africa

A recent study performed on Ethiopian schoolchildren pointed out an *H. pylori* prevalence rate of 65.7% [59]. Comparable prevalence was reported by other studies from rural Ethiopia [60,61]. Another study also assessed schoolchildren, from Kassala city in east Sudan, found a much lower prevalence of *H. pylori*, only 21.8%, compared to the previously mentioned areas [62]. A similar incidence was encountered in children aged between 6 months and 15 years from Nigeria [63]. Nevertheless, the prevalence in Nigeria was reported to differ depending on the geographical area. Thus, Ikpeme et al. [64] reported a prevalence of 30.9% in children from South-South Nigeria, whereas a much higher prevalence was noticed by Senbanjo et al. [65] and Hacombe et al. [66] in those from Lagos (63.6%) and Maiduguri (82%), the latter proving that most of them are infected between 5 and 10 years of age. A systematic review that compared the worldwide distribution of *H. pylori* prevalence found Africa to be the continent with the highest rate of this infection, presenting a prevalence of 70.1%, followed closely by South America (69.4%) and Western Asia (66.6%) [13]. In fact, the authors of this meta-analysis concluded that Nigeria has the highest *H. pylori* prevalence worldwide, at 87.7%. In Southern Africa, Walker et al. [67] reported an overall prevalence of 75%, whereas, in Northern Africa, Bounder et al. [68] found a prevalence of 92.6% in asymptomatic Moroccans and 89.6% in patients complaining of gastric symptoms regardless of age. Another study performed in Egypt revealed a lower prevalence in the general population of only 54.4% [69]. A study from Kenya pointed out a higher prevalence of *H. pylori* infection in children compared to adults, 73.3% versus 54.8% [70]. Another pediatric study from Uganda reported a prevalence of 44.3% in healthy children aged 0–12 years [71]. The data from Ethiopia showed an overall pooled prevalence of 52.2%, with the highest prevalence in Somali (71%) and the lowest in Oromia (39.9%) [72]. Asymptomatic children from Ghana had the lowest prevalence of *H. pylori* infection compared to other reports from Africa, at only 14.2% [73] (Table 1).

Similar to the US, older age was associated with increased odds of positive *H. pylori* status, with the 10–14 age group displaying a stronger association when compared to the 5–9 age group [59]. These findings support the previous results of Lin et al., who found a higher prevalence in children aged between 13–15 years, 12.3%, compared to 11% in those between 9–12 years [74]. Therefore, increasing age might be considered a risk factor for *H. pylori* in African children also [63]. Nevertheless, a study from South-South Nigeria found a lower seroprevalence rate (30.9%), stating that the highest rate was in children aged 6–10 years (40.7%) [75]. According to two studies performed in Sudan and Uganda [62,71], boys are more likely to be infected with *H. pylori* than girls. Schacher et al. found that the most important household-level risk factor for this infection consisted in having a traditional pit or no toilet at all, with a 3.93-fold higher odds of acquiring *H. pylori* infection [59]. Surprisingly, the same authors concluded that having cigarette smokers in the household was a significant protective factor against *H. pylori* infection, decreasing the odds of infection by 68%. A plausible explanation might consist in the effect of nicotine on the gastric mucosa resulting in an increase in gastric acidity and mucosal atrophy and therefore contributing to the auto-eradication of *H. pylori* infection [76]. Certain vegetables, such as Ocimum gratissimum, Carica papaya, and Allium consumed especially in the South-East and South-South of Africa, were also discovered to have a protective effect against *H. pylori* infection [27,46]. A study performed on Nigerian children indicated a great difference in terms of *H. pylori* prevalence between those living in poverty (50%) and those with higher incomes (14.3%) [63]. Birth order was also proved to be a risk factor for *H. pylori* infection in African children since it was highlighted that the first child born has a risk of 13% while the third has a 33.3% chance to develop this infection, with a decrease to 20% in the fourth born child [63]. Contrariwise, this study failed in proving an association between both household size and bed-sharing, and *H. pylori* infection [63]. Nevertheless, according to a pediatric study performed on Nigerian children, the most important risk factors for acquiring *H. pylori* infection were household population, low social class, source of drinking water, household waste disposal used, and type of convenience [75]. In adults, farming profession was also significantly associated with increased *H. pylori* prevalence in Ghana [77] (Table 1). 

In terms of symptoms, schoolchildren from Africa detected with *H. pylori* infection most commonly complain of nausea (25.5%) and epigastric pain (24.5%) [62]. In addition, the same authors found that most children with *H. pylori* were underweight, but *H. pylori* might be considered a confounding factor since malnutrition is a common nutritional disorder in African children regardless of *H. pylori* status. In terms of diagnostic methods, most of the reported studies in Africa used serology testing, which might not be the most accurate mirror of the real prevalence in this population [78]. For example, most of the studies in Libia relied on serological tests for diagnosing *H. pylori* infection [68,69,70]. Nevertheless, in areas like Algeria, Tunisia, West, East, or Central Africa, studies also reported the use of more accurate methods such as a urea breath test, stool antigen, rapid urease test, or histological exam based on upper digestive endoscopy collected gastric biopsies and their combination [79,80,81]. Moreover, the use of certain diagnostic methods may be limited in Africa by poor health infrastructure, poor standard of living, and the limited number of health-care professionals [79]. Another major problem that might contribute to the increased prevalence of this infection in Africa is the lack of guidelines addressing *H. pylori* treatment [78]. 

## 4. *H. pylori* Peculiarities in Asia

An ancient study performed on schoolchildren from Northern Jordan found an increased *H. pylori* prevalence of up to 55.5% in this age group [82]. Based on the findings reported in a recent study (2022) performed on the same population, this prevalence presented a severe decrease (14.6%) when compared to the reports from 2006 [83]. Moreover, the rate reported by Altamimi et al. in the previously published study was much lower compared to neighboring countries [84,85,86]. Although Jordan is considered a low-risk area for gastric cancer with a prevalence of 3.9 per 100,000 population, a recent study performed on Jordanian adults pointed out a very high prevalence of *H. pylori* infection (89%) [87]. These findings might suggest that if the infection is not acquired during childhood, it might not be associated with an increased risk of carcinogenesis, it being well-documented that the process of carcinogenesis requires a considerable amount of time. Based on these facts, we emphasize once more the crucial impact of early diagnosis and treatment in pediatric patients infected with *H. pylori*. At the same time, a study performed on Iraqi children identified a prevalence rate of 27% in young children, with a fast increase up to 58% until the age of 18 years [84]. Reports from Saudi Arabia stated that *H. pylori* infected almost one-third of children below the age of 10 years [85]. More recent data revealed that the seroprevalence of *H. pylori* in healthy Saudi children was 40% [88]. The reported prevalence rate in healthy adults living in the same area presents wide variations, between 23% and 67% [89,90,91,92,93,94,95,96]. In symptomatic patients from Saudi Arabia complaining of dyspepsia and abdominal pain, as well as those undergoing endoscopic procedures, the reported prevalence rates vary between 50–80% [85,97,98,99,100,101,102]. A study performed on symptomatic children from Yemen found a 65% prevalence of *H. pylori* infection [103]. Nevertheless, a recent study from Nepal pointed out a lower overall prevalence of only 16% [104]. Still, 75% of the children below 10 years of age included in the study were detected with positive *H. pylori* stool antigen. Moreover, Mehata et al. recently found an *H. pylori* prevalence of 18.2% in children aged 6–59 months old from Nepal [14]. Mahalanabis et al. stated a similar prevalence (84%) in children aged 6–9 from Bangladesh [105]. Contrariwise the colonization rate in Iran was reported to be as high as 42.7% [106]. In fact, a more recent systematic review performed in Iran revealed a wide variation in *H. pylori’s* overall prevalence, from 30.6–82% [107]. Another study on the Iranian population found a pooled prevalence of 54%, with 42% in children concluding that more than half of this population was infected by *H. pylori* during the last decades [108]. A study performed in Malaysia, which stratified the population according to races living in this area, concluded that Chinese and Indians have the highest prevalence rate of *H. pylori* infection (40–50%) compared to 10–20% in native Malays [109]. Another study performed on children from China pointed out a prevalence of 18.6% [110]. Similar prevalence was also identified by Zhou et al. in Chinese schoolchildren aged 7–12 years (24.1%) [111]. These findings are enigmatic since, despite the increased *H. pylori* prevalence, Indians, unlike the Chinese, have a much lower gastric cancer incidence of less than 10 per 100,000 per year [109]. Lapidot et al. assessed *H. pylori* infection and intestinal microbiome in healthy Israeli children aged between 6–9 years and identified 57% of the participants to be positive for *H. pylori* infection [112]. Japan studies revealed a decrease in the prevalence of this infection from 72.7% in 1974 to approximately 40% in 2014 [113] and 18.8% in 2019 [114]. Park et al. proved in a study from Korea that the prevalence of *H. pylori* in children and young adults decreased considerably, from 60–85% in 1994 to 12.5–28.9% in 2015 [115] (Table 1). 

Several risk factors were reported by different studies in Asia regarding *H. pylori* infection. In terms of age, a study performed on Yemen children concluded that the most affected age group was between 6–8 years [103]. Contrarywise, the male gender was found to predominate in the Iranian population [108]. Thus, younger age seems to be a risk factor for *H. pylori* in children from Asia [103,116,117]. Unsurprisingly lower income was identified as the main risk factor for this infection in children from Asia, although not all studies found a significant association [83,87]. Similar to other reports worldwide, the male gender seems to be a risk factor for *H. pylori* infection also in Jordanian children [82,83,87]. Contrariwise, a study performed in Yemen found that girls were more frequently infected than boys [103], as was identified by Ansari et al. in Nepal [104] and two other studies from China [118,119]. Parental education was suggested as a possible risk factor as well [120], but not all studies sustain this statement [83]. Moreover, Talaiezadeh et al. revealed that an individual’s lower education level represents a risk factor for *H. pylori* colonization [121]. Surprisingly, living in an urban area was discovered to be a significant risk factor in Jordan [83], Vietnam [122], and Nepal [123]. Likewise, according to Ceylan et al., 25.8% of Turkish children living in urban areas were detected with *H. pylori* infection [124]. Recent studies from Jordan revealed that water source was no longer found to represent a harmful effect in terms of *H. pylori* infection [83,87], emphasizing the local improvements in terms of sanitation and accessibility to safe water sources [125]. Thus, lower income and educational level, house crowding, rural residence, the use of tanks as water supplies, alcohol drinking, active smoking, eating spicy food or raw uncooked vegetables, as well as poor living conditions and sanitation represent the most important risk factors for *H. pylori* in children from Saudi Arabia [88,95]. A study performed in Nepal suggested that tea consumption might significantly limit the colonization of *H. pylori* [104]. Dietary habits were also proven to contribute to the limitation of this colonization, being underlined that more than twice meals a day significantly reduced *H. pylori* colonization [104]. Ethnicity seems to be a major risk factor in Asia based on the results of a study from Malaysia, which divides the population depending on the three major Asian races living in this country and identifies Chinese and Indian people as having a significantly higher *H. pylori* prevalence rate as compared to Malays. However, their migration occurred over two generations before [109]. This major finding reveals that in terms of infection prevalence, these people follow the patterns of their country of origin rather than their residence. Recent research pointed out a significant association between low socioeconomic status, *H. pylori* infection, and intestinal bacterial composition [112]. The recently outlined decrease in *H. pylori* infection prevalence in Japan sustains once more the importance of improvements in sanitation and living conditions [114]. The same trend of decreasing prevalence was also noticed in Chinese children, and the most important factors associated with *H. pylori* infection in this population were male gender, age, gastrointestinal symptoms, and having a family member infected [110] (Table 1).

The discrepancies between the reported prevalence of *H. pylori* in different studies are mostly related to the diagnostic method. For example, in terms of Jordan, the study which reported a lower prevalence used a urea breath test for detecting *H. pylori* infection [83], while the older study which found a very high prevalence used serology [82]. Similarly, a recent study performed on Saudi children also based its reports only on serology and encountered a seroprevalence of 40% when assessing both IgM and IgG antibodies, which considerably dropped to 18% if the authors considered only IgG in their analysis [88] emphasizing once more the importance of the screening method. A peculiarity in diagnostic tests was revealed by a systematic review performed by Salto et al., who noticed that the urine antibodies test is used only in Japan [126]. The diagnosis is even more difficult since most children present unspecific symptoms [82,83]. 

## 5. *H. pylori* Peculiarities in Europa

A decrease in *H. pylori* infection prevalence was reported over the last decades in the more affluent countries of Europe [127,128,129], resulting in a further major decrease in gastric cancer incidence and related mortality [30,130,131]. It is worth mentioning that Portugal has the highest gastric cancer incidence in the European Union, 19.2 per 100,000 males and 9.2 per 100,000 females leading to high mortality rates, 15 per 100,000 men and 6.8 per 100,000 females [132,133]. A study performed on adolescents aged 13–17 in Portugal revealed an increased prevalence even at 13 (66.2%), which persisted high throughout adolescence [134]. Similar rates were reported by other studies in Portugal, which found a prevalence of 39.3% in children between 3–14 years of age and 51.5% in those aged 11–15 years [135]. In Bulgarian children, a recent study pointed out an overall *H. pylori* infection prevalence of 24.5%, 2.5-fold lower than the rate reported in previous studies [136]. A study from Germany showed a lower prevalence of 6.5% without significant changes compared to previous reports [137]. Nevertheless, studies performed in the Netherlands [138,139,140], Norway [141], Finland [142,143], the United Kingdom [128,144], and Denmark [145] also revealed a descending trend in *H. pylori* infection. In fact, the Netherlands was reported to have the lowest prevalence of *H. pylori* infection in children aged 1–17 years [146]. According to Mana et al. [147], in Belgium, the prevalence of *H. pylori* infection ranged between 3.2% in children with Belgian parents and 60% in those with foreign parents originating from high prevalence countries. A large study in the Czech Republic reported an overall prevalence of *H. pylori* of 23.5%, with only 4.8% in children aged 15 or less [148] (Table 1). 

Age at acquisition of *H. pylori* infection represents an important trigger for *H. pylori*-associated gastric outcomes [149]. Thus, increased prevalence at younger ages was proved to be associated with a higher risk of gastric cancer as compared to the situation of similar frequency of this infection in older subjects with later acquisition [134,150]. Contrariwise, a study on Bulgarian children did not reveal any significant association between age and *H. pylori* infection [136]. Similar to most studies worldwide, the male gender was also reported to be a risk factor in European children [151]. Nevertheless, a recent study from Romania indicated that females were more prone to develop *H. pylori*-associated gastritis when compared to males in a cohort of children [152]. However, teenage boys were more likely to develop ulcers than females, probably due to higher exposure to certain environmental risk factors like alcohol, tobacco, or ulcerogenic drugs [151]. The same study suggested that gastro-esophageal and duodenogastric reflux might represent protective factors against *H. pylori* infection [136]. Similar findings were reported by Meliț et al., in a Romanian cohort of children [4]. Studies from Romania pointed out that a rural area was associated with an increased risk of acquiring *H. pylori* infection [21,152]. A study performed in Poland, a country with increased *H. pylori* prevalence, concluded that unhygienic behavior, including eating unwashed fruits and vegetables but also not washing hands after school, represents important risk factors for *H. pylori* infection [153]. Nevertheless, another study from this country failed to identify an association between these factors and *H. pylori* infection but instead revealed a significant association between parental education and smoking [134]. The same authors suggested that the number of siblings is relevant only during childhood but not during adolescence. In Germany, *H. pylori* infection was closely dependent on low parental education and unemployment, foreign nationality (having at least one parent birth outside Germany) and having two or more siblings [137]. Aside from low socioeconomic status, a study from Denmark suggested that short duration of education might also be a significant risk factor for acquiring *H. pylori* infection [154]. Smoking was also proved to increase the risk of *H. pylori* infection acquisition [134]. Childcare might also be considered a risk factor for *H. pylori* infection [134]. Moreover, mother-to-child and grandmother-to-child transmission represent an important mechanism in *H. pylori* spread [155]. Iron deficiency and small height for age might be associated with *H. pylori* infection [134]. According to Hirsch et al. [156], dental plaque and root canals might be considered a reservoir of *H. pylori* serving as a potential source of transmission. Marital status seems to also influence the acquisition of *H. pylori* infection according to a study from Czech Republic which concluded that the prevalence of this infection was higher in married, divorced, and widowed individuals [148] (Table 1). 

In terms of symptoms, a Romanian study pointed out that loss of appetite and epigastric pain were significantly associated with *H. pylori* infection in children [21]. Similarly, Rosu et al. also pointed to epigastric pain as the most common symptom in Romanian children with *H. pylori* infection [152]. In fact, Kori et al. stated that abdominal pain and dyspepsia represented the most common indication of endoscopy in European children [151]. This study found that children living in Northern/Western Europe had 4 times higher risk for developing peptic ulcers than those living in Southern Europe. According to Pourakbari et al. [157], the rapid urease test and histology have accuracy comparable to a polymerase chain reaction on biopsies and the stool antigen. Regarding the rapid urease test, its sensitivity in children increases if three or more biopsy samples are assessed or if it is used in older children [158]. The Entero-test is a noninvasive diagnostic method consisting of a small plastic capsule attached to a 90 cm string made of absorbent material, which after being swallowed with water dissolves in the stomach, leaving a string meant to collect gastric juices that may harbor *H. pylori*. It might also represent a reliable noninvasive method for detecting this bacterium [159,160]. Nevertheless, the choice of the diagnostic method should rely on several other criteria, such as the epidemiology of the geographical area, ethnicity, or previous treatments [15]. Finland implemented a ‘screen and treat’ projects which resulted in a considerable decrease in *H. pylori* prevalence [161]. Moreover, a study performed in Belgium revealed that the detection of *H. pylori* relies more and more on histology from gastric biopsies, indicating progress in terms of *H. pylori* diagnosis [148].

**Table 1:** The peculiarities of *H. pylori* infection among continents.

Continents	Prevalence of *H. pylori* Infection	Risk Factors	Observations
**North, Latin & South America**	62.8% before the year 2000 to 60.2% after this period in Latin America and the Caribbean [13] 57.57% Latin America and the Caribbean [27] 59.3% Zamani et al. Latin America and the Caribbean [28] 75% in Alaska [33,34] 59.6%—35–38% in Mexico [40,41,42,43,44] 30% in Brazil [47] rate of gastric cancer 8.7/100,000 in men in Caribbean America, 8.1/100.000 men in Central America, versus 12.7/100.000 men in South America; 5.1, 6.3 and 6.9 per 100,000 in women in Latin America and the Caribbean [25]	persistence of infection → having more than three children in the household [47] using well water instead of municipal water in the United States of America [47] not properly following the recommendations regarding the testing before and after the therapy in the United States of America [52] not use the most accurate test for diagnosing *H. pylori* infection [52] male and poor adherence or difficult access to treatment in Latin America and the Caribbean [49,50,51] the lack of in-home water service might represent a major risk for *H. pylori* reinfection in Alaska [48]	serology should be confirmed by a second test [15,55] gastric biopsy → the most commonly used diagnostic method in more than 50% of the individuals, followed by stool antigen in 20% [48] urea breath test reaches up to 100% for diagnosing *H. pylori* infection in Brazilian children [57]
**Africa**	70.1% for Africa [13] 65.7% in schoolchildren from Ethiopia [59] 21.8% in Sudan [62] 30.9% in the south of Nigeria [65] 54.4% in Egypt [69] children versus adults, 73.3% versus 54.8% in Kenya [70] 44.3% in healthy children aged between 0–12 years in Uganda [71] asymptomatic children → 14.2% in Ghana [73]	higher prevalence in children aged between 13–15 years [74] boys are more likely to be infected with *H. pylori* as compared to girls in Uganda and Sudan [62,71] having a traditional pit or no toilet at all, with a 3.93-fold higher odds of *H. pylori* infection [59] poverty in Nigerian children birth order (the first child born has a risk of 13% while the third has a 33.3% chance to develop this infection, with a decrease to 20% in the fourth born child [63]) household population, low social class, source of drinking water, household waste disposal used, and type of convenience [75] in adults, farming profession in Ghana [77]	cigarette smokers in the household—protective factor against *H. pylori* infection [76] vegetables such as Ocimum gratissimum, Carica papaya, and Allium—protective effect against *H. pylori* infection [27,46] schoolchildren from Africa most commonly complain of nausea (25.5%) and epigastric pain (24.5%) [62] in Libia—serological tests most used for diagnosing *H. pylori* infection [68,69,70] Algeria, Tunisia, West, East or Central Africa—use of more accurate methods [79,80,81]
**Asia**	55.5% in schoolchildren from Jordan [82] 89% for adults in Jordan [87] low-risk area for gastric cancer with a prevalence of 3.9 per 100,000 in Jordan [87] 27% in young children, with a fast increase up to 58% in those aged between 2 and 18 years in Iraq [84] 1/3 of the children below <10 years in Saudi Arabia [85] healthy Saudi children 40% [88] 23–67% in adults in Saudi Arabia [89,90,91,92,93,94,95,96] 50–80% in symptomatic patients in Saudi Arabia [85,97,98,99,100,101,102] symptomatic children → 65% in Yemen [103] 16% in Nepal [104] 75% of the children below 10 years of age in Nepal [14] (84%) in children aged 6–9 years from Bangladesh [105] 30.6–82% in Iran [107] Chinese and Indians—the highest prevalence rate of *H. pylori* infection (40–50%) as compared to 10–20% in native Malays [109] Indians—gastric cancer incidence of less than 10 per 100,000 per year [109] children → 18.6% in China [110] schoolchildren aged 7–12 years in China (24.1%) [111] Israeli healthy children → 57% [112] prevalence decrease from 72.7% in 1974 to approximately 40% in 2014 [113] and 18.8% in 2019 in Japan [114] decreased considerably from 60–85% in 1994 to 12.5–28.9% in 2015 in Korea [115]	male gender was found to predominate in Jordan [82,83,87] living in an urban area was identified to be a significant risk factor in Jordan [83], Vietnam [122], and Nepal [123] girls were more frequently infected than boys in Yemen [103] girls were more frequently infected in Nepal [104] male gender was found to predominate in Iranian population [108] ethnicity [109] girls were more frequently infected in China [118,119] lower income—a main risk factor, although not all studies found a significant association in China [83,87] male gender in adults, age, gastrointestinal symptoms, and having a family member infected in China [110] most affected age group was between 6–8 years in Yemen [103] lower income and educational level, house crowding, rural residence, the use of tanks as water supplies, alcohol drinking, active smoking, eating spicy food or raw uncooked vegetables, poor living conditions, and sanitation in children from Saudi Arabia [88,95]	tea consumption might significantly limit the colonization of *H. pylori* in Nepal [104] more than twice meals a day significantly reduced the *H. pylori* colonization in China [104] lower prevalence if urea breath test used, but higher with serology—Jordan [83] Saudi children had a seroprevalence of 40% when assessing both IgM and IgG antibodies, which considerably dropped to 18% if only used [88] urine antibodies test in Japan [126]
**Europe**	age of 13 (66.2%) and remained high throughout adolescence in Portugal [134] 39.3% in children between 3–14 years of age and 51.5% in those aged 11–15 years [135] 24.5% in Bulgarian children [136] 6.5% in children from Germany [137] the prevalence of *H. pylori* infection ranged between 3.2% in children with Belgian parents and 60% in those with foreign parents originating from high prevalence countries [147] 23.5% overall prevalence in Czech Republic, with only 4.8% in children aged 15 or less [148] Portugal—the highest gastric cancer incidence in the European Union, 19.2 per 100,000 males and 9.2 per 100,000 females; mortality rates, 15 per 100,000 men and 6.8 per 100,000 females [132,133] studies in Netherlands [138,139,140], Norway [141], Finland [142,143], United Kingdom [128,144], and Denmark [145], descending trend in *H. pylori* infection	age an important trigger for *H. pylori*-associated gastric outcomes [149] male gender → a risk factor in European children [151] females → more frequently infected in Romania [152] teenage boys more likely to develop ulcers [151] rural area associate with increased risk in Romania [21,152] eating unwashed fruits and vegetables, and not washing hands after school are risk factors in Poland [153] low parental education and unemployment, foreign nationality (having at least one parent birth outside Germany), and having two or more siblings in Germany [137] short duration of education in Denmark [154] higher in married, divorced, and widowed individuals in the Czech Republic [148]	mother-to-child and grandmother-to-child transmission represent an important mechanism in *H. pylori* spread [155] dental plaque and root canals—reservoir of *H. pylori* serving as a potential source of transmission [156] prevalence at younger ages associated with higher gastric cancer [134,150] duodenogastric reflux → protective factors against *H. pylori* infection [4,136] symptoms in Romanian—loss of appetite and epigastric pain were significantly associated with *H. pylori* infection in children [21,152] abdominal pain and dyspepsia—the most common indication for endoscopy in children from Europe [151] the rapid urease test and histology have comparable accuracy to polymerase chain reaction on biopsies and the stool antigen [157] Enterotest might also represent a reliable noninvasive method [159]

## 6. Concluding Remarks and Future Perspectives 

The prevalence of *H. pylori* infection has wide variations worldwide, and it is extremely important to assess the factors that contribute to these discrepancies. To elucidate the accurate contribution of risk factors within each geographic area, further studies should focus more on studying the differences related to the environmental factors, diagnostic methods, eradication regimens, and host-related peculiarities. Thus, the identification of major risk factors, especially in areas with increased *H. pylori* infection prevalence, might represent the cornerstone for effective and long-term eradication, which will further result in decreasing gastric cancer incidence worldwide. Future perspectives should focus more on assessing the host’s genetic susceptibility and immune response to the infection to design more accurate diagnostic methods and more effective therapeutic regimens. The challenges related to the diagnosis of *H. pylori* infection are considerable and own a major negative impact on the long-term persistence of this infection since false-negative results are not uncommon in pediatric patients. Thus, these challenges are either related to the procedure itself since invasive methods are usually refused by the parents or are difficult to perform in small children or to the relatively lower sensitivity and specificity of noninvasive tests, which are preferred in this age group. Personalized medicine should represent a future approach in terms of both diagnosis and treatment of pediatric *H. pylori* infection to decrease the life-threatening events related to this infection that might occur during adulthood, such as gastric cancer, but also other more controversial disorders like stroke, cardiovascular disease, diabetes, or autoimmune disorders. Elucidating the role of gastric and gut microbiota should also represent a future target in terms of *H. pylori* infection due to its great implications for the successful eradication rate, especially since probiotics were proven to increase this rate if added to standard eradication regimens. The peculiarities of this infection in low-prevalence countries should represent a valuable lesson for countries where the prevalence is very high or has an ascending trend. Nevertheless, further studies should be performed to more precisely detect all the mechanisms involved in the differences regarding *H. pylori* infection prevalence worldwide.

## Data Availability

Not applicable.
